# Cellular and Molecular Mechanisms of Wound Repair: From Biology to Therapeutic Innovation

**DOI:** 10.3390/cells14231850

**Published:** 2025-11-24

**Authors:** Caijun Jin, Yongxun Jin, Zhiyuan Ding, Kong Srey Nuch, Mira Han, JungHee Shim, Pham Ngoc Chien, Chan Yeong Heo

**Affiliations:** 1 Department of Plastic and Reconstructive Surgery, College of Medicine, Seoul National University, Seoul 03080, Republic of Korea; jcjking96@snu.ac.kr (C.J.); jinyongxun789@snu.ac.kr (Y.J.); jiwon4@snu.ac.kr (Z.D.); sreynuchkong123@snu.ac.kr (K.S.N.); 2 Department of Plastic and Reconstructive Surgery, Seoul National University Bundang Hospital, Seongnam 13620, Republic of Korea; 99506@snubh.org (M.H.); xmylife@empas.com (J.S.)

**Keywords:** wound healing, molecular mechanisms, cellular mechanism

## Abstract

Wound repair preserves tissue integrity through four overlapping phases—hemostasis, inflammation, proliferation, and remodeling—coordinated by platelets, neutrophils, macrophages, fibroblasts, keratinocytes, endothelial cells, and stem/progenitor cells acting with growth factors, chemokines, extracellular matrix, and intracellular signaling. Disruption of these programs results in chronic non-healing wounds or fibrotic scarring. Recent work delineates microbial influences, epigenetic and transcriptomic regulation, and cellular heterogeneity resolved by single-cell and spatial omics. Concurrent advances in biomaterials, engineered scaffolds, stem cell-derived products, and genome-targeted approaches are enabling mechanism-based therapies. Persistent challenges include wound heterogeneity, systemic modifiers such as diabetes and aging, and safe, effective delivery of biologics. This review summarizes cellular and molecular mechanisms of cutaneous repair, outlines deviations that underlie pathological healing, and evaluates emerging concepts and translational strategies. Integrating classical models with contemporary insights supports the development of precision wound medicine and personalized interventions to improve outcomes and quality of life.

## 1. Introduction

The restoration of tissue integrity following injury is a fundamental biological imperative for multicellular organisms [1]. In humans, wound repair represents a highly complex and dynamic process that integrates hemostatic responses, immune defense, cellular proliferation, and tissue remodeling into a coordinated sequence of events [2]. This orchestration involves a diverse array of cell types and molecular mediators that function within a temporally and spatially regulated framework. While the overall process is evolutionarily conserved and remarkably robust, its failure or dysregulation results in pathological outcomes that have significant clinical and socioeconomic consequences [3]. Chronic non-healing wounds, such as diabetic foot ulcers, venous stasis ulcers, and pressure sores, affect millions of individuals worldwide and impose a substantial financial burden on healthcare systems [4,5]. Conversely, excessive or aberrant repair responses can give rise to hypertrophic scars, keloids, or organ fibrosis, conditions that may impair function and remain therapeutically challenging [6].

The cellular landscape of wound repair is highly heterogeneous. Platelets initiate the process through clot formation and the release of growth factors that recruit inflammatory cells [7]. Neutrophils and macrophages dominate the inflammatory phase, mediating pathogen clearance, debris removal, and the regulation of subsequent reparative responses [8]. Fibroblasts and their differentiated progeny, myofibroblasts, are central to extracellular matrix (ECM) deposition and wound contraction, while keratinocytes drive re-epithelialization through coordinated proliferation and migration [9]. Endothelial cells contribute to angiogenesis, ensuring adequate nutrient and oxygen delivery to regenerating tissues [10]. In parallel, stem and progenitor cell populations, both resident and bone marrow–derived, provide additional cellular plasticity that supports regeneration [11].

At the molecular level, wound repair is governed by a complex milieu of soluble mediators, matrix components, and intracellular signaling cascades [12]. Growth factors such as vascular endothelial growth factor (VEGF), platelet-derived growth factor (PDGF), fibroblast growth factors (FGFs), and transforming growth factor-β (TGF-β) act as master regulators of angiogenesis, fibroblast activation, and epithelial regeneration [13]. Cytokines and chemokines, including interleukin-1 (IL-1), interleukin-6 (IL-6), tumor necrosis factor-α (TNF-α), and monocyte chemoattractant protein-1 (MCP-1), orchestrate inflammatory cell recruitment and polarization [14]. Concurrently, ECM remodeling enzymes such as matrix metalloproteinases (MMPs) and their tissue inhibitors maintain a balance between matrix degradation and deposition [15]. These extracellular cues converge on intracellular pathways including MAPK, PI3K/AKT, NF-κB, and Wnt/β-catenin, which regulate cellular survival, proliferation, migration, and differentiation [16].

In recent years, advances in experimental biology have provided unprecedented insights into wound repair mechanisms. High-resolution imaging, single-cell transcriptomics, and spatial multi-omics approaches have uncovered cellular heterogeneity and lineage dynamics with remarkable detail [17]. The influence of the microbiome on cutaneous wound healing has emerged as an important area of investigation, highlighting the interplay between host immunity and microbial communities [18]. Epigenetic regulation and non-coding RNAs have been recognized as critical modulators of wound-associated gene expression, offering novel therapeutic targets [19]. Parallel progress in biomaterials, bioengineered scaffolds, and cell-based therapies has begun to translate mechanistic understanding into innovative strategies for clinical intervention [20].

This review synthesizes current knowledge on the cellular and molecular mechanisms of wound repair. We begin by outlining the canonical phases of healing, followed by an in-depth discussion of the roles of distinct cell populations and molecular mediators. We then examine pathological deviations from normal repair, including chronic wounds and fibrotic scarring, and highlight emerging concepts that are reshaping the field. Finally, we consider the therapeutic implications of these insights and the challenges that remain in translating them into effective, patient-specific treatments. By integrating classical knowledge with contemporary advances, this review aims to provide a comprehensive framework for understanding wound biology and to guide future directions in both basic and translational research.

## 2. Phases of Wound Healing

**Figure 1:**
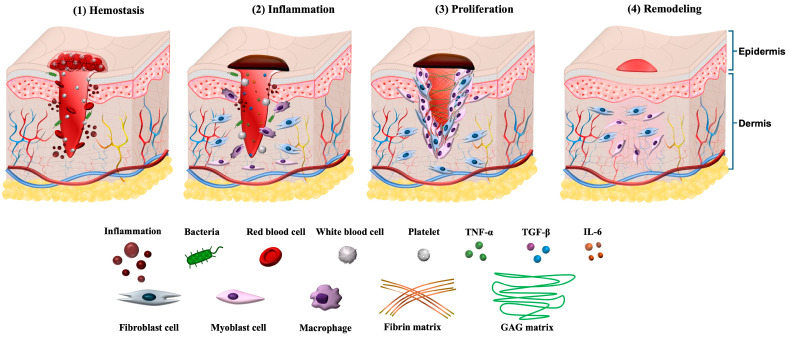
Illustration of four phases in the wound healing process. (1) Hemostasis: Platelet aggregation and fibrin clot formation seal the wound. (2) Inflammation: Neutrophils and macrophages clear debris and initiate immune signaling. (3) Proliferation: Keratinocyte migration, fibroblast activation, ECM deposition, and angiogenesis restore tissue structure. (4) Remodeling: Collagen fiber reorganization and scar maturation occur. Reprinted from Ref. [21].

Wound healing is traditionally divided into four overlapping but distinct phases: hemostasis, inflammation, proliferation, and remodeling (Figure 1). These phases represent coordinated biological programs that collectively restore tissue continuity and function. While the temporal sequence is conserved, the duration and quality of each phase can vary depending on the type of injury, the tissue microenvironment, and systemic host factors such as age, comorbidities, and metabolic status. The success of repair relies on precise regulation of these phases and the effective transition from one stage to the next.

### 2.1. Hemostasis

The earliest response to tissue injury is the rapid activation of hemostatic mechanisms that prevent blood loss and establish a provisional matrix for subsequent cellular infiltration [22]. Vascular disruption triggers platelet adhesion, aggregation, and degranulation, leading to the formation of a fibrin-rich clot [23]. Activated platelets release a broad spectrum of bioactive mediators, including PDGF, VEGF, and TGF-β, which serve as chemoattractants for neutrophils, monocytes, and fibroblasts [24]. In addition to their hemostatic role, platelets function as early regulators of repair by providing a scaffold that supports cell migration and by delivering signals that initiate the inflammatory phase [25].

### 2.2. Inflammation

The inflammatory phase is characterized by the recruitment and activation of innate immune cells [26]. Neutrophils are among the first responders, arriving within hours of injury [8]. They exert antimicrobial functions through the production of reactive oxygen species, proteolytic enzymes, and neutrophil extracellular traps, while also clearing cellular debris [27,28]. Their activity, though essential, must be tightly regulated to avoid excessive tissue damage. Monocytes subsequently infiltrate the wound and differentiate into macrophages, which orchestrate the transition from inflammation to repair. Early macrophages adopt a pro-inflammatory M1-like phenotype, producing cytokines such as TNF-α, IL-1β, and IL-6 [29]. As healing progresses, macrophages shift toward an anti-inflammatory and reparative M2-like phenotype, secreting interleukin-10 (IL-10), growth factors, and matrix-remodeling enzymes [30]. This polarization is critical for resolving inflammation and initiating the proliferative phase.

### 2.3. Proliferation

The proliferative phase is dominated by the generation of new tissue to replace that lost or damaged by injury. Fibroblasts, recruited by PDGF and TGF-β, proliferate and differentiate into myofibroblasts, producing ECM proteins such as collagen type I and III, fibronectin, and proteoglycans [31]. This ECM provides structural integrity and a substrate for further cell migration [32]. Concurrently, keratinocytes at the wound edge migrate and proliferate to restore epithelial continuity, a process facilitated by growth factors including epidermal growth factor (EGF) and keratinocyte growth factor (KGF) [33]. Angiogenesis, mediated by endothelial cells in response to VEGF and FGFs, ensures adequate oxygen and nutrient supply to the regenerating tissue [34]. The proliferative phase culminates in the formation of granulation tissue, characterized by abundant capillaries, fibroblasts, and a loose ECM.

### 2.4. Remodeling (Maturation)

The final phase of wound repair involves the maturation and remodeling of granulation tissue into a more stable and functional scar. Myofibroblasts contract the wound margins, reducing wound size [35]. Collagen fibers undergo reorganization and crosslinking, with a gradual replacement of type III collagen by the stronger type I collagen, resulting in increased tensile strength [15]. MMPs and their inhibitors regulate ECM turnover, ensuring the removal of excess matrix and preventing fibrosis [36]. Vascular regression occurs as metabolic demands decline, leaving behind a relatively avascular scar [37]. This phase may last for weeks to months, and although structural continuity is restored, the regenerated tissue rarely achieves the full strength and functionality of uninjured skin.

## 3. Cellular Players in Wound Repair

The successful progression of wound healing depends on the coordinated actions of multiple cell types that interact dynamically within the injured tissue microenvironment. Each cellular population contributes distinct yet overlapping functions, ranging from clot formation and immune defense to ECM deposition, angiogenesis, and re-epithelialization. Their activities are regulated by a diverse network of growth factors, cytokines, chemokines, and matrix-derived cues. Understanding the specific roles of these cellular players is essential for elucidating the mechanisms of normal repair and for identifying targets to correct pathological healing. Table 1 provides a summary of each major cell type’s role in wound healing, including key mediators, pathways, model evidence, and recent references.

### 3.1. Platelets

Platelets are the first cellular responders at the site of injury. Platelets initiate hemostasis and lay a fibrin scaffold that recruits and instructs reparative cells [38]. Through degranulation, platelets release PDGF, VEGF, TGF-β, and EGF, which (i) drive angiogenesis by stimulating endothelial migration, proliferation, and vessel stabilization; (ii) activate fibroblast chemotaxis, proliferation, collagen synthesis, and myofibroblast differentiation to build granulation tissue; and (iii) enhance MSC homing and lineage commitment to replenish tissue-specific cells [7]. In parallel, platelet chemokines and antimicrobial peptides such as platelet factor 4 (CXCL4/PF4), C-X-C motif chemokine ligand 7 (CXCL7), defensins and thrombocidins, modulate innate immunity and host defense, supporting infection control during early repair [39]. Recent studies revealed that platelet-derived extracellular vesicles act as critical mediators of intercellular communication during wound repair, delivering growth factors, microRNAs, and cytokines that promote angiogenesis, re-epithelialization, and tissue regeneration while modulating inflammation and reducing scar formation [40]. Additionally, platelets can transfer functional mitochondria to mesenchymal stem cells via clathrin-mediated endocytosis, activating citrate metabolism and de novo lipogenesis to boost VEGF and hepatocyte growth factor (HGF) secretion and enhance angiogenesis and wound healing [41]. In conclusion, their activity bridges the hemostatic and inflammatory phases, creating both a physical scaffold and a biochemical environment conducive to repair. Autologous platelet-rich plasma is used to promote healing in various wounds and tissues in numerous clinical scenarios [42,43].

### 3.2. Neutrophils

Neutrophils are the first immune cells recruited to injury sites, arriving within hours via gradients of hydrogen peroxide, formyl peptides, and chemokines such as interleukin-8 (CXCL8/IL-8) and leukotriene B_4_ (LTB_4_), which amplify recruitment through CXC chemokine receptor 1/2 (CXCR1/2) and Leukotriene B_4_ receptor 1 (BLT1) signaling [44]. Once activated, they perform phagocytosis, release reactive oxygen species (ROS), and secrete proteases such as elastase and MMP-9 that degrade necrotic tissue and pathogens, thereby debriding the wound bed and facilitating subsequent cell migration [45,46]. Neutrophils also produce chemokines and growth factors such as, VEGF [47], HGF [48], IL-1β, and TNF-α [49] that attract macrophages and fibroblasts, linking innate defense to tissue repair [50].

However, if neutrophil activation persists, excessive ROS and protease release damage the extracellular matrix and endothelial cells, prolong inflammation, and impair re-epithelialization. Resolution of neutrophil activity occurs through apoptosis followed by macrophage efferocytosis, which shifts macrophages toward an anti-inflammatory (M2-like) phenotype that promotes granulation and angiogenesis [51,52]. Notably, a subset of neutrophils can undergo reverse migration away from the wound, potentially contributing to systemic inflammation [53,54].

Neutrophils also form extracellular traps (NETs) that immobilize microorganisms [55]. NETs exert a dual role in wound repair [56]. While transient NETs formation aids antimicrobial defense in early wounds, excessive or persistent NETs, as observed in diabetic and chronic wounds, induce tissue damage, amplify inflammation, and impair angiogenesis [56]. Proteases and ROS associated with NETs disrupt the extracellular matrix and endothelial integrity, thereby delaying healing. Targeting NET formation or promoting their clearance has emerged as a potential therapeutic strategy for refractory wounds [57,58]. A recent study demonstrated that excessive accumulation of NETs in diabetic wounds impaired angiogenesis and delayed repair by inducing endothelial-to-mesenchymal transition (EndMT) via the TLR-9/PAK2–Merlin/NF2 suppression of Hippo-YAP signaling [59]. In vivo, inhibition of NETosis or mothers against decapentaplegic homolog 2 (SMAD2) silencing reduced EndMT, restored angiogenesis, and accelerated wound closure, identifying the NETs–EndMT axis as a key mechanism and therapeutic target in diabetic wound healing [59]. However, the extent to which EndMT is reversible in vivo remains uncertain; it is unclear whether endothelial cells that undergo mesenchymal transition can revert to their original state once the fibrotic stimulus is removed.

### 3.3. Macrophages

Macrophages are central orchestrators of wound healing, mediating the transition from inflammation to repair. Initially, monocyte-derived macrophages adopt a pro-inflammatory phenotype, producing TNF-α, IL-1β, and IL-6 to amplify immune responses and clear debris [60]. As the wound progresses, macrophages undergo phenotypic switching toward an anti-inflammatory, reparative state characterized by secretion of IL-10, VEGF, and TGF-β [61]. These reparative macrophages promote angiogenesis, fibroblast activation, and ECM remodeling. The plasticity of macrophages is a defining feature of their function, and defects in their polarization are strongly associated with impaired healing and chronic wounds [62]. Recently, Li Tao et al. found that interleukin-27 (IL-27), downregulated in diabetic skin, restored early M1 responses and subsequently promoted the M1 to M2 transition via the IL-27–STAT3 axis, thereby enhancing collagen deposition and angiogenesis [63]. By contrast, Shiyan Li et al. found that interleukin-25 (IL-25) directly drove M2 polarization through the PI3K/AKT/mTOR pathway and simultaneously activated fibroblasts via TGF-β/SMAD signaling, boosting ECM accumulation and angiogenesis [64]. Together, these findings highlight that sequential modulation of macrophage polarization may represent a promising therapeutic strategy. However, the M1/M2 classification is an oversimplification. In reality, macrophages exhibit a continuum of activation states, and determining how to optimally shift or balance these states in vivo remains an open question.

### 3.4. Fibroblasts and Myofibroblasts

Fibroblasts are central regulators of cutaneous repair, orchestrating both tissue regeneration and scar formation through their heterogeneity, plasticity, and dynamic responses to the wound microenvironment [9]. Distinct fibroblast lineages and anatomical subpopulations exert divergent functions [65]. Engrailed-1 lineage–positive (CD26^+^) fibroblasts are closely associated with fibrotic scarring [66], whereas Engrailed-1 lineage–negative fibroblasts tend to support regenerative outcomes [67].

During repair, fibroblasts contribute through two major functional modules. First, phenotypic conversion into myofibroblasts, driven by TGF-β/Smad and c-Jun signaling, promotes wound contraction and ECM deposition [68,69]; persistent activation, however, leads to hypertrophic scarring [70]. Second, fascia-derived fibroblasts migrate collectively into the wound bed, not only generating early contractile forces but also delivering preassembled ECM “patches” that accelerate wound closure [71]. This process depends on cell–cell adhesion and communication molecules, such as *N*-cadherin and Connexin 43, and complements the classic granulation tissue–myofibroblast paradigm [72,73].

Fibroblasts also act as mechanosensitive and immunomodulatory hubs. Through integrin- and DNA damage response (DDR)-mediated activation of FAK–YAP/TAZ pathways, they sense matrix stiffness, while interacting with type 2 immune signaling, such as interleukin-33 (IL-33), interleukin-4 (IL-4), and interleukin-13 (IL-13) to shape the inflammatory-to-repair transition [74,75,76,77]. Notably, inhibition of FAK or YAP reduces scarring and enhances regenerative features, including hair follicle neogenesis [67,78].

Additionally, recent research using time-resolved single-cell and spatial transcriptomic profiling found that fibroblasts exhibit the most dynamic temporal and spatial gene-expression patterns, transitioning from early edge- and Prg4^+^ fibroblasts to late-stage Col25a1^+^/Crabp1^+^ and Pamr1^+^/Ly6a^+^ fibroblast subpopulations that spatially stratify the scar and regulate immune–stromal signaling [79]. This finding provides a comprehensive cellular atlas of fibroblast heterogeneity, offering new insight into how temporal–spatial fibroblast dynamics govern scar architecture and wound repair outcomes.

Collectively, these findings underscore fibroblasts as multifaceted effectors of wound healing, integrating lineage identity, phenotypic plasticity, mechanical cues, and immune crosstalk to determine whether a wound heals with regeneration or fibrosis.

### 3.5. Keratinocytes

Keratinocytes are essential for re-epithelialization, the process by which the epidermal barrier is restored [33]. Following injury, keratinocytes at the wound edge undergo phenotypic changes that enable migration over the provisional matrix. This is facilitated by growth factors such as EGF and KGF, as well as integrin-mediated adhesion to ECM components [80]. Proliferation of basal keratinocytes replenishes the migrating population, while differentiation restores stratification of the epidermis. Based on this, an increasing number of studies are now exploring the roles and functions of keratinocytes in wound healing [81]. Holt et al., using live-cell spatiotemporal imaging, found that dynamic enrichment or activation of the mechanosensitive channel PIEZO1 at keratinocyte wound edges drives localized retraction that slows collective migration and delays re-epithelialization, whereas PIEZO1 deletion accelerates closure [82]. Additionally, recent research also found that epidermal autophagy in keratinocytes promotes wound healing in vivo by driving C–C motif chemokine ligand 2 (CCL2) transcription through an AMPK–BRAF–ERK–AP-1 pathway, thereby coordinating keratinocyte proliferation, migration, and dermal fibroblast activation [83]. These studies further elucidate the molecular mechanisms by which keratinocytes regulate wound healing, providing new insights and potential strategies for promoting effective tissue repair.

Keratinocytes also serve as immune sentinels during wound healing by recognizing damage- and pathogen-associated signals through TLR and cGAS–STING pathways, leading to the release of IL-1, IL-6, IL-8, MCP-1, CCL20 and antimicrobial peptides that recruit and regulate neutrophils, macrophages, and T cells [84]. They can also present antigens via major histocompatibility complex class II (MHC-II), and their inflammatory output is finely tuned by epigenetic modulators such as JMJD3 and microRNA, such as miR-132, miR-19a/b and miR-20a [85,86,87,88]. The disruption of this regulation results in prolonged inflammation and delayed healing. Additionally, recent research found that the expression of lncRNA SNHG26 in keratinocytes drives the inflammatory-to-proliferative state transition by interacting with ILF2 to divert it from inflammatory gene loci, such as JUN, IL6, IL8 and CCL20 to LAMB3, and that loss of SNHG26 impairs re-epithelialization and exacerbates inflammation in vivo [89].

Additionally, the latest study using single-cell and spatial transcriptomics of human skin wounds revealed a precisely timed cellular program: migratory keratinocytes, macrophages, and neutrophils dominate the early phase, shifting by day 7 to proliferative keratinocytes and fibroblasts that drive re-epithelialization and matrix formation, and by day 30 to remodeling fibroblasts and angiogenic niches [90]. Notably, keratinocyte-derived EGFR ligands and macrophage-secreted EREG/CXCL1/CXCL5 coordinate FOSL1-dependent keratinocyte migration, whereas chronic ulcers exhibit failed migration due to persistent inflammation.

### 3.6. Endothelial Cells

Endothelial cells drive angiogenesis, a hallmark of the proliferative phase. In response to hypoxia and VEGF signaling, endothelial cells sprout from pre-existing vessels, migrate into the wound bed, and form new capillary networks [10,91]. These nascent vessels deliver oxygen and nutrients necessary for tissue regeneration and provide a route for leukocyte trafficking. Endothelial cells also secrete paracrine factors that modulate fibroblast, keratinocyte and other cells’ behavior [92,93,94]. Angiogenesis is transient, and capillary density declines during remodeling as vascular regression stabilizes the scar tissue.

Ferroptosis has emerged as a key mechanism linking endothelial dysfunction to impaired wound repair. Jin et al. showed that Piezo1-mediated Ca^2+^ influx activates the CaMKII/ATF3 pathway, represses SLC7A11, and drives ferroptosis in senescent endothelial cells, thereby inhibiting angiogenesis and delaying healing [95]. Inhibition or genetic silencing of Piezo1 or ATF3 alleviated ferroptosis and markedly improved wound closure in aged mice.

Endothelial cells can undergo EndMT during wound repair, losing endothelial markers such as VE-cadherin and CD31, while gaining mesenchymal traits such as vimentin, *N*-cadherin, and α-SMA [96]. Transient EndMT supports angiogenesis and vascular remodeling, but its persistence contributes to fibrosis and scar formation by driving myofibroblast differentiation and excessive ECM deposition [96]. Key regulators include TGF-β, FGF-2, hypoxia, ROS, and inflammatory cytokines, converging on transcription factors like Snail, Slug, Twist, and Zeb1.

### 3.7. Stem and Progenitor Cells

Resident and circulating stem or progenitor cells provide additional regenerative capacity [97,98]. MSCs, derived from bone marrow, adipose tissue, and dermis, contribute to repair by differentiating into fibroblast-like cells and by secreting trophic factors with immunomodulatory and angiogenic properties [99,100,101]. Endothelial progenitor cells participate in angiogenesis, while epidermal stem cells located in hair follicles and basal epidermis sustain keratinocyte replenishment [102,103]. The therapeutic potential of stem and progenitor cells has been highlighted in preclinical and clinical studies, though challenges remain in harnessing their full regenerative capacity. Especially, recent advances have introduced a growing number of stem cell–based or cell-free therapeutic strategies combined with bioactive carriers, such as hydrogels and other biomaterial scaffolds, which effectively reprogram macrophage polarization, activate fibroblasts via TGF-β/SMAD3, and enhance angiogenesis to promote wound healing [104,105,106].

**Table 1:** Major Cell Types Involved in Cutaneous Wound Healing and Their Functional Roles.

Cell Type	Primary Role in Wound Healing	Key Growth Factors/Cytokines Involved	Evidence (In Vitro/In Vivo/Ex Vivo)	Representative Signaling Pathways	Reference
Platelets	Initiate hemostasis and provide early pro-healing signals; release mediators that recruit immune and stromal cells.	PDGF, VEGF, TGF-β, EGF, PF4	In vivo: mouse skin wound models; PRP clinical applications.	MAPK/ERK; PI3K/AKT; TGF-β/Smad	[107,108,109]
Neutrophils	Early antimicrobial defense, debris clearance, and inflammatory mediator release; excessive activity delays healing.	IL-1β, TNF-α, MPO, NETs-associated proteins	In vivo: acute and diabetic wound models; ex vivo: human wound exudates.	NF-κB; ROS-associated pathways; TLR signaling	[110,111,112]
Macrophages	Orchestrate transition from inflammation to repair; polarization from M1→M2 regulates ECM deposition and angiogenesis.	TNF-α, IL-6 (M1); IL-10, TGF-β, VEGF (M2)	In vivo: macrophage depletion and lineage tracing studies; ex vivo: human ulcer biopsies.	NF-κB (M1), STAT3/STAT6 (M2), PI3K/AKT	[113,114,115]
Fibroblasts/Myofibroblasts	Produce and remodel ECM; myofibroblasts contract wound tissue; lineage subtypes influence fibrosis vs. regeneration.	TGF-β1, CTGF, Collagen I/III, Fibronectin	In vivo: lineage tracing and scRNA-seq of wound fibroblasts; in vitro: fibroblast activation assays.	TGF-β/Smad; YAP/TAZ mechanotransduction; FAK–c-Jun axis	[79,116,117]
Keratinocytes	Re-epithelialization through migration and proliferation serves as an immune sentinel.	EGF, KGF, IL-1, IL-6, CCL20	In vitro: scratch-wound migration assays; in vivo: re-epithelialization kinetics in murine wounds.	EGFR/MAPK; Integrin–FAK; cGAS–STING signaling	[118,119]
Endothelial Cells	Drive angiogenesis and restore perfusion; transient EndMT supports vascular remodeling.	VEGF, Ang-1/2, FGF-2	In vivo: angiogenesis markers in wound beds; ex vivo: vascular sprouting assays.	VEGF/VEGFR2; Notch–DLL4; HIF-1α oxygen-sensing pathways	[120]
Stem/Progenitor Cells	Provide regenerative capacity, paracrine immunomodulation, and enhance angiogenesis and ECM remodeling.	VEGF, HGF, IL-10, SDF-1/CXCL12	In vivo: MSC transplantation models; Clinical: cell-based wound therapies	PI3K/AKT; TGF-β/Smad3; CXCL12/CXCR4 axis	[121,122]

## 4. Molecular Mediators and Pathways in Wound Repair

**Figure 2:**
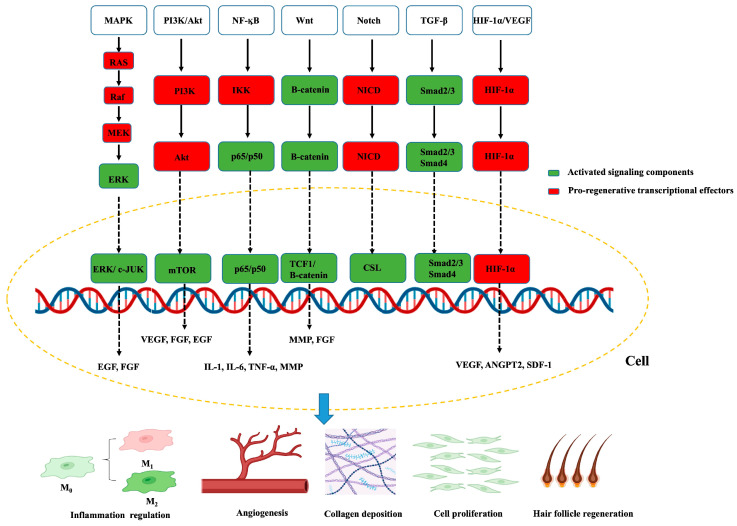
Key intracellular signaling pathways involved in wound healing. Red boxes indicate activated signaling components; green boxes denote transcriptional effectors promoting tissue regeneration. Arrows indicate direct activation steps, whereas dashed arrows denote nuclear translocation and transcriptional regulation. The yellow dashed circle outlines the cellular context in which pathways operate, with convergence at the level of gene transcription. The lower schematic links these pathways to major cellular outcomes, including macrophage polarization, angiogenesis, collagen deposition, fibroblast proliferation, and hair-follicle regeneration. Created by the authors.

Wound repair is orchestrated by a complex and dynamic interplay of soluble mediators, ECM components, and intracellular signaling pathways. These molecular cues regulate cell migration, proliferation, differentiation, and survival across the different phases of healing. The balance between pro-inflammatory and anti-inflammatory mediators, growth-promoting and inhibitory signals, and matrix deposition and degradation is critical for the restoration of tissue integrity (Figure 2). Dysregulation of these molecular mechanisms underlies pathological healing outcomes, including chronic non-healing wounds and excessive fibrosis.

### 4.1. Growth Factors

Growth factors are central regulators of wound repair and act through autocrine, paracrine, and juxtacrine mechanisms [123]. PDGF recruits neutrophils, macrophages, and fibroblasts to the wound site and stimulates fibroblast proliferation and ECM production [124]. VEGF is a potent inducer of angiogenesis, promoting endothelial cell proliferation, migration, and new capillary formation [125]. FGFs, including FGF-2, facilitate fibroblast activation, keratinocyte proliferation, and angiogenesis [126,127]. TGF-β plays a dual role, stimulating fibroblast-to-myofibroblast differentiation and collagen deposition while also modulating immune responses [128]. EGF and KGF are crucial for re-epithelialization [129,130]. Together, these growth factors provide the essential cues for tissue regeneration.

Although growth factors can effectively stimulate keratinocyte and fibroblast proliferation, angiogenesis, and matrix remodeling, their clinical application is limited by short half-life, enzymatic degradation, and poor local retention in wound environments [131]. Emerging solutions pair these biologics with engineered delivery systems, such as hydrogels, ECM-mimetic scaffolds, micro or nanoparticles, and microneedles, to prolong local retention, enable spatiotemporal release, and reduce dose and toxicity [132,133,134,135]. Additionally, complementary protein engineering, such as ECM-binding fusions, PEGylation, or protease-resistant, further enhances stability and potency at the wound site [136,137,138,139]. Together, these strategies are shifting growth factor therapy from high-dose, burst exposure toward controlled, multi-factor regimens better aligned with the wound’s hemostasis–inflammation–proliferation–remodeling timeline.

### 4.2. Cytokines and Chemokines

Cytokines and chemokines regulate the inflammatory environment of the wound. Pro-inflammatory cytokines such as TNF-α, IL-1, and IL-6 are produced predominantly by neutrophils and macrophages during the early inflammatory phase [14]. They enhance leukocyte recruitment, vascular permeability, and antimicrobial defense. As repair progresses, anti-inflammatory cytokines such as IL-10 and TGF-β suppress excessive inflammation and promote resolution [102,140,141,142].

Chemokines play essential and temporally coordinated roles in cutaneous wound healing by orchestrating leukocyte recruitment, angiogenesis, and tissue remodeling [143]. In the early inflammatory phase, ELR^+^ CXC chemokines, such as CXCL1, CXCL2, CXCL5, CXCL6, and CXCL8, recruit neutrophils and initiate angiogenesis through CXCR1/2 signaling [144,145,146], while CC chemokines, such as CCL2, CCL3, and CCL5, attract monocytes and macrophages to promote the transition toward the proliferative phase [147,148,149,150,151]. During tissue regeneration, CXCL12–CXCR4 signaling guides progenitor cell migration and granulation tissue formation [152], whereas CXCL10–CXCR3 mediates re-epithelialization and capillary pruning during remodeling [153,154,155]. Additionally, CCL2 and CXCL12 orchestrate the recruitment of monocytes, lymphocytes, and stem/progenitor cells into the wound bed [156,157,158,159]. The coordinated activity of cytokines and chemokines ensures a controlled inflammatory response that transitions toward tissue regeneration.

### 4.3. Intracellular Signaling Pathways

Multiple intracellular signaling cascades integrate extracellular cues to coordinate cellular responses during healing. The MAPK/ERK pathway integrates growth-factor and integrin cues, such as EGF/FGF–RTKs, to coordinate keratinocyte and fibroblast proliferation, directed migration, and endothelial sprouting [160,161,162]. The PI3K/AKT pathway promotes cell survival, angiogenesis, and metabolic adaptation in hypoxic wound environments [163,164]. In addition to modulating the expression of growth factors such as VEGF, FGF, and EGF, activation of the PI3K/AKT signaling pathway facilitates cellular proliferation and migration, angiogenesis, and collagen biosynthesis [165,166]. The NF-κB pathway, upon activation by injury or pathogen-derived cues, induces innate immune mediators, such as pro-inflammatory cytokines, chemokines, and adhesion molecules, supports keratinocyte proliferation and migration, and MMP expression to enable re-epithelialization [167,168]. The Wnt/β-catenin signaling is a central driver of re-epithelialization and granulation, promoting keratinocyte proliferation and migration, fibroblast activation, and angiogenic support through β-catenin–TCF-dependent transcription and MMP [169,170]. During wound healing, FGF9 activates the Wnt signaling cascade in fibroblasts, which in turn induces FGF9 expression to reinforce Wnt activity through a positive feedback mechanism that drives hair follicle regeneration [171,172]. The Notch signaling pathway modulates angiogenesis and epithelial differentiation [173,174,175]. These pathways exhibit extensive cross-talk, ensuring robust yet adaptable regulation of wound repair.

### 4.4. Extracellular Matrix Remodeling

The ECM provides both structural support and biochemical signals that regulate wound repair. During the proliferative phase, fibroblasts and myofibroblasts secrete collagen, fibronectin, and proteoglycans to establish granulation tissue [176,177]. ECM remodeling is tightly regulated by MMPs and their endogenous inhibitors, tissue inhibitors of metalloproteinases (TIMPs) [178,179]. MMPs facilitate keratinocyte migration by degrading the provisional matrix and allow angiogenic sprouting by remodeling basement membranes [180]. Dysregulated ECM turnover, either excessive degradation or excessive deposition, can result in chronic wounds or fibrotic scarring, respectively [15]. ECM components also function as signaling reservoirs, releasing bioactive fragments, such as matrikines that influence cell adhesion, migration, and proliferation [181,182].

### 4.5. Immune-Metabolic Crosstalk

Metabolic regulation has emerged as a critical determinant of wound repair. Hypoxia-inducible factor-1α (HIF-1α) mediates cellular adaptation to hypoxic conditions by promoting angiogenesis and glycolysis with upregulation of VEGF, ANGPT2, SDF-1-CXCR4-mediated endothelial progenitor cell homing, metabolic adaptation, and keratinocyte or fibroblast programs for re-epithelialization and granulation [183,184,185]. Glucose metabolism influences macrophage polarization, with glycolytic metabolism favoring pro-inflammatory phenotypes and oxidative metabolism supporting reparative functions [186,187]. Lipid mediators, including prostaglandins and leukotrienes, further regulate inflammation and tissue remodeling [188,189]. The integration of immune and metabolic signals ensures that energy-demanding reparative processes are matched to available resources in the wound microenvironment [190].

## 5. Pathological Wound Healing

While wound healing is generally efficient under physiological conditions, disturbances in the cellular and molecular mechanisms can result in pathological outcomes. These may manifest as impaired healing, characterized by chronic non-healing wounds, or excessive healing, leading to fibrosis and abnormal scarring. Both conditions impose a significant clinical and economic burden and underscore the importance of precise regulation of repair processes.

### 5.1. Chronic Non-Healing Wounds

Chronic wounds, including diabetic foot ulcers, venous stasis ulcers, and pressure ulcers, exemplify impaired healing in which wounds remain stalled in a persistent inflammatory state [191]. They represent a pathological breakdown of the coordinated wound-healing cascade, characterized by sustained inflammation, disrupted intercellular communication, and defective tissue remodeling [192,193]. At the molecular level, chronic ulcers exhibit prolonged NF-κB activation, elevated ROS, and excessive protease activity, which degrade growth factors and extracellular matrix components essential for re-epithelialization [194,195,196]. Keratinocytes in these wounds display hyperproliferation but reduced migratory capacity, while fibroblasts become senescent and lose responsiveness to TGF-β, resulting in diminished collagen synthesis and aberrant ECM deposition [197,198]. Macrophage polarization remains skewed toward a pro-inflammatory M1-like phenotype, delaying the resolution phase and angiogenic signaling [199]. Concurrently, endothelial cells exhibit impaired VEGF responsiveness and reduced angiogenesis, leading to local hypoxia and nutrient deprivation [200,201]. Multi-omics and spatial transcriptomic analyses further reveal disorganized immune-stromal communication and metabolic dysregulation, particularly in diabetic and venous ulcers [202,203,204]. Additionally, biofilms are a principal driver of chronic, non-healing wounds by forming polymicrobial, ECM-embedded communities that resist antibiotics and immune clearance, sustain low-grade inflammation, and degrade tissue, thereby delaying re-epithelialization and granulation [205,206]. Collectively, these molecular and cellular abnormalities lock the wound into a chronic inflammatory state, preventing the progression to proliferation and remodeling, and underscore the necessity of precision-targeted, stage-specific interventions to restore coordinated healing.

### 5.2. Fibrosis and Hypertrophic Scarring

Pathological scarring reflects a breakdown of phase transitions in normal repair, with wounds failing to resolve inflammation and instead entering a pro-fibrotic immune milieu [207]. The cardinal features of fibrosis and hypertrophic scar are that expansion of pro-fibrotic immune cells, such as M2-polarized macrophages, dendritic cells, mast cells, and Th2 lymphocytes, amplifies TGF-β1 signaling, which drives fibroblast-to-myofibroblast differentiation, excessive type I/III collagen deposition, and matrix stiffening [208,209,210]. This immune–stromal axis explains how sustained NF-κB-biased inflammation, ROS and protease excess, and degraded growth-factor bioavailability prevent progression from the inflammatory to the proliferative/remodeling phases, locking tissue into a feed-forward fibrotic loop that maintains myofibroblast survival and contractility [6]. Clinically, fibrosis and hypertrophic scar thus emerge as an aberrant wound-healing phenotype in which delayed re-epithelialization and disordered matrix turnover coexist with persistent myofibroblasts [211]. Accordingly, therapeutic control of inflammation and TGF-β1 activity is highlighted as a rational strategy to reduce pathological scarring. Nonetheless, because TGF-β1 is also crucial for normal tissue repair, completely blocking its activity may impair healing. Thus, there is an unresolved need to balance TGF-β’s pro-regenerative effects against its pro-fibrotic effects. The other studies further link mechanical tension and matrix stiffness to myofibroblast activation and scar persistence, reinforcing the concept that immune cues and mechanotransduction cooperate to determine scar outcomes [212,213]. Together, these insights position hypertrophic scar as dysregulated wound healing driven by maladaptive immune programs and TGF-β–centered fibroblast activation, with opportunities for intervention at the inflammation → TGF-β1 → myofibroblast axis and its crosstalk with matrix mechanics.

### 5.3. Systemic and Environmental Influences

**Figure 3:**
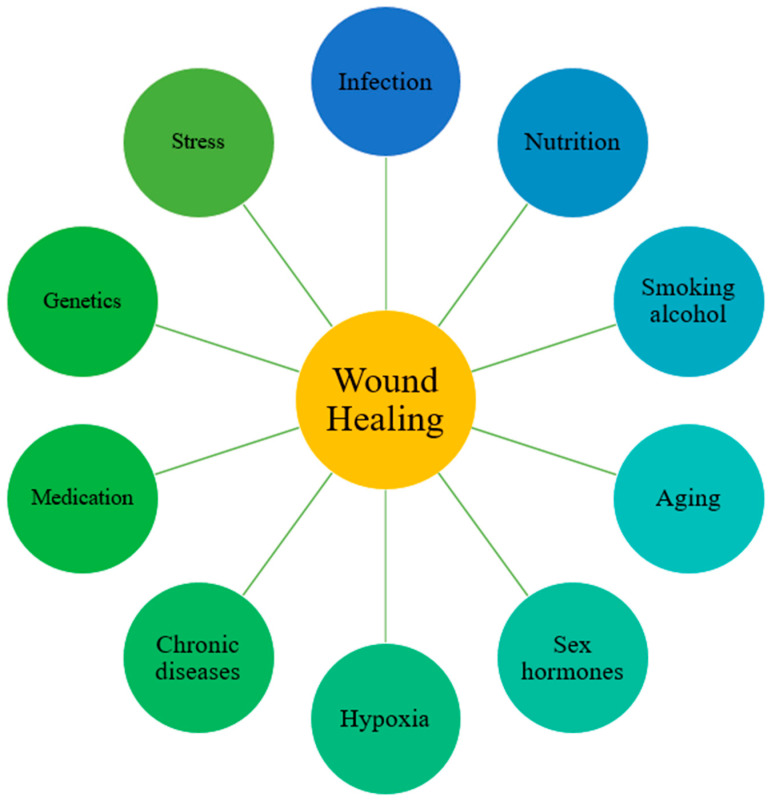
Factors that affect wound healing. Common situations that delay skin wound healing. Reprinted from Ref. [224].

Pathological healing is also shaped by systemic conditions and environmental factors (Figure 3). Advanced age is characterized by cellular senescence and reduced responsiveness to growth factors, resulting in delayed re-epithelialization and extracellular matrix deposition [214]. Malnutrition, particularly deficiencies in protein and micronutrients, compromises fibroblast activity and collagen synthesis, thereby weakening tissue repair [215,216]. Vascular disorders such as atherosclerosis and diabetes impair tissue perfusion, leading to chronic hypoxia and nutrient deprivation that delay granulation and re-epithelialization [217]. Immunosuppression caused by systemic diseases or pharmacological agents prolongs inflammation and limits regenerative capacity by reducing immune cell function [218]. Cigarette smoking induces vasoconstriction and cytotoxic injury, diminishing oxygen delivery and impairing angiogenesis and fibroblast activity [219]. Persistent infection, especially by biofilm-forming bacteria, maintains excessive protease and cytokine levels that degrade growth factors and extracellular matrix components, arresting progression to the proliferative phase [220,221]. Sustained hypoxia further suppresses cellular proliferation and collagen production [222]. Genetic predisposition also contributes, as individuals susceptible to keloid or hypertrophic scarring exhibit exaggerated collagen deposition and prolonged inflammation, predisposing them to pathological healing outcomes [223]. Thus, pathological wound healing emerges from a convergence of local cellular and molecular dysregulation and systemic host factors [16].

### 5.4. Clinical Implications

The dual extremes of pathological healing, impaired regeneration, and excessive fibrosis illustrate the delicate balance required for optimal repair. Therapeutic strategies aimed at correcting these imbalances include growth factor supplementation, cytokine modulation, stem cell–based interventions, and anti-fibrotic agents targeting TGF-β signaling. Despite advances, effective clinical management remains challenging, highlighting the need for therapies tailored to the underlying cellular and molecular defects.

## 6. Emerging Concepts and Technologies in Wound Repair

Recent advances in experimental biology, systems medicine, and bioengineering have expanded the understanding of wound healing beyond classical frameworks. These developments have uncovered previously unrecognized layers of complexity, including the role of commensal microbes, epigenetic regulation, cellular heterogeneity, and the influence of engineered biomaterials. Together, these emerging concepts provide novel insights into wound biology and open new avenues for therapeutic innovation.

### 6.1. The Microbiome in Wound Healing

The skin microbiome, once regarded primarily as a potential source of infection, is now recognized as an important regulator of cutaneous repair [18]. Commensal microorganisms can influence immune homeostasis by modulating Toll-like receptor signaling and cytokine production in keratinocytes and resident immune cells [225,226]. In chronic wounds, microbial dysbiosis characterized by reduced microbial diversity and overrepresentation of pathogenic species such as Staphylococcus aureus or Pseudomonas aeruginosa is associated with persistent inflammation, biofilm formation, and impaired epithelialization [227,228]. The therapeutic modulation of microbial communities, through targeted antibiotics, probiotics, or microbiome-based therapies, represents an emerging strategy to restore balanced host–microbe interactions and promote healing [229,230].

### 6.2. Epigenetic and Transcriptomic Regulation

Epigenetic mechanisms, including DNA methylation and demethylation, histone modifications and chromatin remodeling, and non-coding RNAs, such as lncRNAs, miRNAs and circRNAs, coordinate the timing and cell-type specificity of repair programs across keratinocytes, fibroblasts, endothelial and immune cells [19]. In keratinocytes, DNMT1 sustains progenitor states while TET2-mediated demethylation of the MMP9 promoter under hyperglycemia or TNFα drives pathological matrix degradation [231,232,233]. lncRNAs such as WAKMAR1 enhance migration by modulating DNA methylation of E2F1, whereas MALAT1 links infection to impaired angiogenic signaling [234,235]. In fibroblasts, HDAC4/6/8 are required for TGF-β1–induced myofibroblast differentiation [236,237], and lncRNA-directed histone methylation, such as H19–EZH2, H3K4me3, can activate HIF-1α pathways to improve closure in diabetes [238]. Macrophage polarization is tuned by writers/erasers, such as MLL1, SETDB2, JMJD3, and SWI/SNF components, determining the switch from inflammatory to reparative states [239,240]. In neutrophils, PAD4-dependent histone citrullination licenses NETosis, which is exaggerated in diabetic wounds [241,242]. MicroRNAs also play critical roles in wound healing by modulating gene expression that controls inflammation, cell proliferation, migration, and tissue remodeling, thereby ensuring the proper transition through each phase of the repair process [243,244]. Collectively, chronic wounds display maladaptive epigenetic profiles, global methylation drift, persistent pro-inflammatory chromatin states, and ncRNA dysregulation that stall the transition to proliferation and remodeling, nominating epigenetic biomarkers and therapeutics, such as HDAC/HAT modulators, DNMT/TET targeting, and lncRNA therapeutics for precision wound care [245,246].

### 6.3. Single-Cell and Spatial Omics Approaches

Traditional bulk analyses mask the heterogeneity of cellular responses within the wound microenvironment [247,248]. Single-cell RNA sequencing and spatial transcriptomics now enable high-resolution mapping of cell types, states, and interactions across the healing timeline [249,250]. These technologies have identified distinct fibroblast and macrophage subpopulations with specialized reparative or pathological roles, as well as previously unrecognized progenitor cells contributing to re-epithelialization and angiogenesis [79,251]. Such approaches not only deepen mechanistic understanding but also facilitate the identification of molecular targets for precision therapies [202].

### 6.4. Biomaterials and Bioengineered Scaffolds

**Figure 4:**
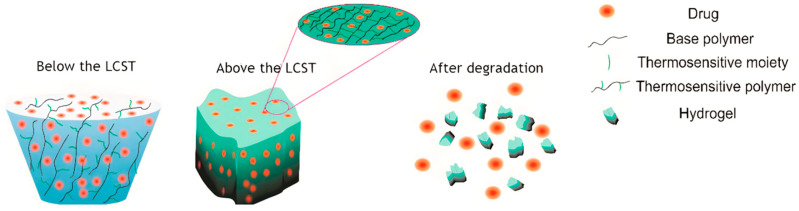
Hydrogel drug delivery system for in situ formation of scaffolds. A schematic depicting a thermo-responsive hydrogel that remains liquid during application but solidifies into a gel upon warming to body temperature, above its Lower Critical Solution Temperature (LCST), forming a scaffold within the wound. Reprinted from Ref. [252].

The field of regenerative medicine has developed biomaterials designed to mimic the extracellular matrix and provide structural and biochemical support for repair. Natural and synthetic scaffolds, hydrogels, and nanofiber matrices have been engineered to deliver growth factors, cytokines, or stem cells in a controlled manner (Figure 4). For example, certain injectable hydrogels are thermo-responsive, remaining liquid at room temperature but forming a gel upon reaching body temperature, above their Lower Critical Solution Temperature (LCST), enabling in situ scaffold formation. Advanced biomaterials can be tailored to degrade at desired rates, modulate immune responses, and promote angiogenesis. Incorporation of bioactive molecules and gene-delivery systems further enhances their therapeutic potential. Clinical translation of these technologies is ongoing, with promising results in the treatment of chronic wounds and burns.

## 7. Therapeutic Implications

Translating mechanistic insights from cellular and molecular studies of wound repair into effective therapies has long been a central goal of regenerative medicine and dermatology. Despite advances in basic science, clinical management of complex wounds remains challenging, and many patients fail to respond to conventional interventions. Current therapeutic strategies, both established and experimental, target different aspects of the healing cascade, including growth factor supplementation, cytokine modulation, stem and progenitor cell delivery, biomaterial-based scaffolds, and anti-fibrotic agents.

### 7.1. Growth Factor–Based Therapies

Recombinant growth factors represent one of the earliest attempts to accelerate wound repair. Topical PDGF-BB (becaplermin, Regranex^®^, 0.01% gel; Smith & Nephew, Inc., Fort Worth, TX, USA) is approved for the treatment of diabetic foot ulcers and has demonstrated moderate efficacy in promoting granulation tissue formation and re-epithelialization [253,254]. VEGF- and FGF-based therapies have shown promise in preclinical models by enhancing angiogenesis, although translation to the clinic has been limited by issues of short half-life, off-target effects, and challenges in controlled delivery [255]. Combination approaches, integrating multiple growth factors or coupling them with delivery matrices, are under investigation to better recapitulate the physiological signaling environment [256,257].

### 7.2. Cytokine and Inflammation Modulation

Targeting cytokine signaling offers opportunities to correct dysregulated inflammation in chronic wounds [258]. Agents that inhibit excessive TNF-α or IL-1 activity may reduce persistent inflammatory states, while IL-10 supplementation has been proposed to promote macrophage polarization toward reparative phenotypes [259,260]. However, systemic administration carries risks of immunosuppression, and localized, temporally controlled delivery remains a major hurdle [261]. Advanced biomaterials capable of releasing cytokines in a controlled and tissue-specific manner may address these challenges [262].

### 7.3. Stem and Progenitor Cell–Based Approaches

Cell-based therapies, including MSC transplantation and endothelial progenitor cell delivery, have demonstrated immunomodulatory and pro-angiogenic effects in preclinical and early clinical studies [11,263,264]. Genetic engineering, including CRISPR/Cas-based approaches, is being explored to enhance the regenerative capacity of therapeutic cells or to correct defective molecular pathways in chronic wounds [265,266]. Viral and non-viral gene delivery systems targeting growth factor expression, such as VEGF or PDGF, are under investigation, though challenges remain regarding safety, efficiency, and regulatory approval [267,268].

### 7.4. Biomaterials and Tissue Engineering

**Figure 5:**
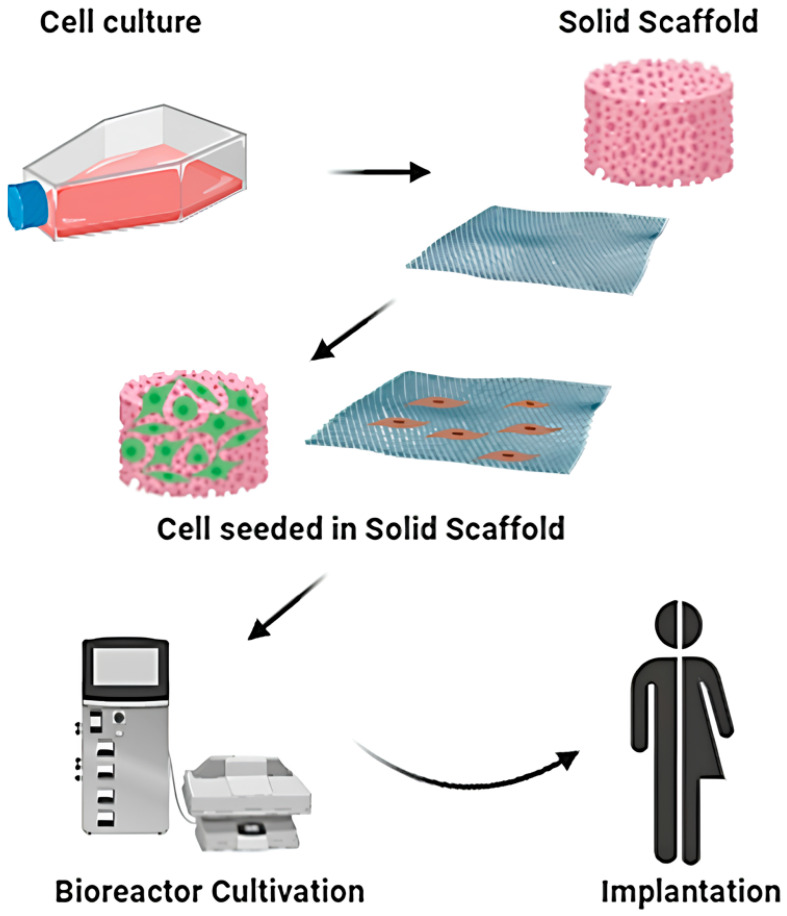
Schematic representation of classical tissue engineering approach. Reprinted from Ref. [273].

Bioengineered scaffolds and hydrogels are increasingly integrated into wound care, providing both structural support and bioactive cues [20]. Traditional tissue engineering involves culturing cells and seeding them onto a solid scaffold, followed by bioreactor cultivation to promote tissue maturation before implantation into the patient (Figure 5). However, with the progress of research, an increasing number of therapeutic approaches based on traditional tissue engineering techniques are being developed. Commercial products such as acellular dermal matrices and collagen-based dressings facilitate cellular infiltration and ECM deposition [269]. Advances in nanotechnology and 3D bioprinting now enable the design of customizable scaffolds capable of delivering growth factors, nucleic acids, or therapeutic cells [270]. Smart biomaterials that respond to environmental stimuli, such as pH or enzymatic activity, are being developed to enable precision delivery and dynamic regulation of the wound microenvironment [271,272].

### 7.5. Anti-Fibrotic Strategies

Preventing or reducing fibrosis remains a critical therapeutic goal in patients predisposed to hypertrophic scars and keloids [9,274]. TGF-β inhibitors, angiotensin II receptor blockers, and modulators of myofibroblast differentiation are under investigation for their potential to suppress excessive collagen deposition [208,275,276]. Emerging therapies targeting epigenetic regulators and non-coding RNAs associated with fibrotic gene expression offer additional strategies [277]. Clinical translation of anti-fibrotic agents requires careful balancing of efficacy with preservation of essential repair processes [278].

### 7.6. Challenges and Opportunities

Although significant progress has been made, several challenges remain in translating molecular and cellular insights into effective wound therapies [279]. The heterogeneity of wounds, influenced by etiology, comorbidities, and patient-specific factors, complicates the development of universal treatments [280]. Delivery of biologics to the wound bed in a stable, controlled, and cost-effective manner remains a major limitation [261]. Moreover, rigorous clinical trials with standardized endpoints are needed to validate emerging therapies, and several recent trials are summarized in Table 2 to illustrate current translational efforts. Precision medicine approaches, integrating omics profiling and systems biology, may enable stratification of patients and tailoring of therapies to individual healing defects [281].

## 8. Future Directions and Unanswered Questions

Despite decades of research, wound healing remains an incompletely understood biological process, and current therapeutic options for pathological wounds are often suboptimal. Advances in cellular and molecular biology have substantially expanded the mechanistic framework of repair, yet several critical questions remain unresolved. Addressing these gaps will be essential to develop next-generation therapies that are both effective and patient-specific.

### 8.1. Integration of Local and Systemic Factors

Most mechanistic studies have focused on local cellular and molecular interactions within the wound bed. However, systemic influences including aging, diabetes, vascular disease, metabolic status, and immune senescence exert profound effects on repair [285]. Future research must bridge the gap between local wound biology and systemic physiology, elucidating how endocrine, metabolic, and immune networks converge to shape outcomes. Multi-organ and whole-body models of repair, coupled with systems-level analyses, are needed to capture these complex interactions.

### 8.2. Heterogeneity of Cellular Responses

Recent single-cell and spatial transcriptomic studies have highlighted the remarkable heterogeneity of fibroblasts, macrophages, endothelial cells, and keratinocytes during repair [248,286]. Yet the lineage relationships, temporal dynamics, and functional significance of many subpopulations remain incompletely defined. Clarifying how specific cell subsets contribute to regeneration versus fibrosis, and how they are regulated by niche signals, will be critical for identifying precise therapeutic targets.

### 8.3. Microbiome and Host–Microbe Interactions

Although dysbiosis is clearly associated with chronic wounds, the causal mechanisms by which microbial communities modulate healing remain poorly defined. Key unanswered questions include how specific microbial taxa influence immune polarization, keratinocyte behavior, and angiogenesis, and whether targeted modulation of the microbiome can reproducibly improve clinical outcomes [287,288]. Integration of metagenomic and metabolomic analyses with host transcriptomics may provide deeper insight into these interactions.

### 8.4. Epigenetic and Non-Coding RNA Regulation

Epigenetic mechanisms have emerged as major regulators of repair, yet the identity of key chromatin modifications, histone marks, and regulatory RNAs that determine successful versus pathological healing is not fully established. It remains unclear whether epigenetic reprogramming can be therapeutically manipulated in a safe and durable manner. Future work should explore the feasibility of targeting non-coding RNAs, DNA methylation, or histone modifications as part of regenerative strategies.

### 8.5. Translation of Regenerative Technologies

Biomaterials, stem cell therapies, and gene-editing technologies hold considerable promise, but translation into clinical practice has been limited. Challenges include immune rejection, variability in cell potency, difficulties in large-scale manufacturing, and concerns about safety and regulatory approval. A major question is how to optimize delivery systems to achieve spatially and temporally controlled therapeutic effects within the dynamic wound environment. Comparative trials and standardized endpoints are needed to assess the efficacy of these interventions.

### 8.6. Toward Precision Wound Medicine

Perhaps the most pressing future direction is the development of precision medicine approaches for wound care [289]. Chronic wounds are heterogeneous, with diverse etiologies and patient-specific risk factors, yet current treatments are often applied uniformly [290]. Integration of multi-omics profiling, advanced imaging, and computational modeling may enable stratification of patients according to molecular signatures and predicted therapeutic responses [291]. Key unanswered questions include how to define actionable biomarkers, how to align these biomarkers with tailored interventions, and how to implement precision approaches in diverse healthcare settings.

## 9. Conclusions

In summary, wound healing is an intricately orchestrated biological process whose disruption leads to chronic wounds or fibrotic scarring. Recent advances in omics technologies, biomaterials, and regenerative medicine have reshaped our understanding of repair and revealed promising therapeutic opportunities. Yet, translating these insights into effective clinical interventions remains challenging due to patient heterogeneity, comorbid conditions, and limitations in targeted delivery. Future progress will rely on the integration of molecular mechanisms with precision and bioengineering approaches through sustained multidisciplinary collaboration, ultimately advancing the development of safe and personalized therapies that restore both the structure and function of injured tissues.

## Figures and Tables

**Table 2:** Recent Clinical Trials on Wound Healing Strategies (2022–Present).

Intervention/Strategy	Phase	Target Condition	Study Design	Outcome Measures	Clinical Trial Numbers	Status/Year	Reference
Botanical hydrogel (Lavior^®^)—a natural extract-based hydrogel for DFU	Phase 2	Diabetic foot ulcers	Randomized non-inferiority trial vs. standard hydrogel	Primary: Ulcer healing rate. Secondary: time to closure, wound size reduction	NCT05607979	Completed (2023)	-
Triticum vulgare (Fitostimoline^®^) hydrogel—polyhexanide and wheat extract for DFU	Phase 4	Diabetic foot ulcers	Randomized controlled trial vs. saline gauze dressing	Primary: Proportion of wounds achieving full healing. Secondary: infection rate, adverse events.	NCT05661474	Completed (2022)	[282]
Autologous adipose-derived stem cells (ASCs) platelet-rich plasma (PRP)	Phase 1	Diabetic foot ulcers	3-arm randomized trial: cultured ASCs + PRP, stromal vascular fraction + PRP, vs. standard care	Primary: Wound closure rate and time to healing; Secondary: safety (adverse events), wound re-epithelialization quality	NCT05610865	Recruiting (2020–2025)	-
Adipose-derived exosome therapy—topical application of adipose exosome product	Observational (Pilot)	Refractory full-thickness skin ulcers	One-arm open-label pilot study	Primary: Percentage area reduction, time to closure. Infection and inflammation	NCT05475418	Completed (2022)	[283]
Adipose derived extracellular vesicles therapy—topical product	Interventional (RCT)	Chronic full-thickness skin ulcers	Multicenter randomized controlled trial	Primary: Incidence of complete wound closure at predefined time point. Secondary: Healing rate, time to closure, adverse events.	NCT06253975	Ongoing (Initiated 2024)	-
Purified exosome product + fibrin sealant (PEP + Tisseel)—topical exosomal biologic combined with fibrin gel	Phase 2a	Diabetic foot ulcers	Multicenter randomized controlled trial	Primary: Wound closure rate at 12 weeks. Secondary: ulcer area reduction, time to 50% healing, incidence of adverse events	NCT04326959	Recruiting (2024)	-
Engineered probiotic (ILP100-Topical)—Limosilactobacillus reuteri modified to secrete CXCL12 for wound repair	Phase 2a	Diabetic foot ulcers	Randomized, double-blind, placebo-controlled trial	Primary: Safety and incidence of target ulcer healing at 5 weeks. Secondary: Percent wound area reduction over 5 weeks, long-term wound recurrence	NCT05608187	Terminated early (2024)	-
hepatocyte growth factor (HGF) gene therapy	Phase 3	Neuroischemic diabetic foot ulcers	Multicenter placebo-controlled Phase III	Primary: Complete ulcer closure rate at 3–5 months. Secondary: Time to wound closure, change in ulcer area, safety, and tolerability.	NCT02563522	Completed (2023)	[284]

## Data Availability

No new data were created or analyzed in this study.

## Abbreviations

The following abbreviations are used in this manuscript:

ECM	Extracellular matrix
VEGF	Vascular endothelial growth factor
PDGF	Platelet-derived growth factor
FGFs	Fibroblast growth factors
TGF-β	Transforming growth factor-β
IL-1	Interleukin-1
IL-6	Interleukin-6
TNF-α	Tumor necrosis factor-α
MCP-1	Monocyte chemoattractant protein-1
MMPs	Matrix metalloproteinases
IL-10	Interleukin-10
EGF	Epidermal growth factor
KGF	Keratinocyte growth factor
MSC	Mesenchymal stem cell
CXCL4/PF4	Platelet factor 4
CXCL7	C-X-C motif chemokine ligand 7
HGF	Hepatocyte growth factor
CXCL8/IL-8	Interleukin-8
LTB_4_	Leukotriene B_4_
CXCR 1	CXC chemokine receptor 1
CXCR 2	CXC chemokine receptor
BLT1	Leukotriene B_4_ Receptor 1
ROS	Reactive oxygen species
NETs	Neutrophils extracellular traps
EndMT	Endothelial-to-mesenchymal transition
TLR-9	Toll-like receptor 9
PAK2	p21-activated kinase 2
NF2	Neurofibromin 2
YAP	Yes-associated protein
SMAD2	Mothers against decapentaplegic homolog 2
IL-27	Interleukin-27
STAT3	Signal transducer and activator of transcription 3
IL-25	Interleukin-25
DDR	DNA damage response
FAK	Focal adhesion kinase
TAZ	Transcriptional co-activator with PDZ-binding motif)
IL-33	Interleukin-33
IL-4	Interleukin-4
IL-13	Interleukin-13
Prg4	Proteoglycan 4
Col25a1	Collagen XXVα1
CRABP1	Cellular retinoic acid-binding protein 1
Pamr1	Peptidase domain containing associated with muscle regeneration 1
Ly6a	Lymphocyte antigen 6a
CCL2	C–C motif chemokine ligand 2
AMPK	AMP-activated protein kinase
BRAF	v-raf murine sarcoma viral oncogene homolog B1
ERK	Extracellular signal–regulated kinase
AP-1	Activator protein-1
cGAS	Cyclic GMP–AMP synthase
STING	Stimulator of interferon genes
MHC-II	Major histocompatibility complex class II
JMJD3	Jumonji domain-containing protein D3
CCL20	C–C motif chemokine ligand 20
LAMB3	Laminin subunit beta 3
EREG	Epiregulin
FOSL1	FOS like 1
CaMKII	Calcium-dependent protein kinase II
ATF3	Activating transcription factor 3
SLC7A11	Solute carrier family 7 member 11
A-SMA	α-smooth muscle actin
Zeb1	Zinc finger E-box binding homeobox 1
SMAD3	Mothers against decapentaplegic homolog 3
ELR	Tripeptide motif Glu–Leu–Arg
RTK	Receptor tyrosine kinase
TCF	T-cell factor
TIMPs	Tissue inhibitors of metalloproteinases
HIF-1α	Hypoxia-inducible factor-1α
ANGTP2	Angiopoietin-2
SDF-1	Stromal cell–derived factor-1
DNMT1	DNA methyltransferase 1
TET-2	Ten-eleven translocation methylcytosine dioxygenase 2
WAKMAR1	Wound and keratinocyte migration associated lncRNA 1
E2F1	E2F transcription factor 1
MALAT1	Metastasis-associated lung adenocarcinoma transcript 1
HDAC	Histone deacetylase
EZH2	Enhancer of zeste homolog 2
H3K4me3	Trimethylation of histone H3 on lysine 4
MLL1	Mixed lineage leukemia 1
SETDB2	SET domain bifurcated 2
SWI/SNF	SWItch/sucrose non-fermentable complex
PAD4	Peptidylarginine deiminase 4
HAT	Histone acetyltransferase
CRISPR/Cas	Clustered regularly interspaced short palindromic repeats/CRISPR-associated system
